# Lenvatinib enhances antitumor immunity of anti-PD-1 antibody

**DOI:** 10.1007/s10147-025-02721-5

**Published:** 2025-02-22

**Authors:** Yu Kato

**Affiliations:** Tsukuba Research Laboratories, Eisai Co., Ltd., Tsukuba, Ibaraki Japan

**Keywords:** Angiogenesis inhibitor, Anti-PD-1 antibody, FGF, Lenvatinib, TAMs, VEGF

## Abstract

Lenvatinib is an orally available multi-tyrosine kinase inhibitor that mainly targets vascular endothelial growth factor (VEGF) and fibroblast growth factor (FGF) signaling. These inhibitory activities of lenvatinib exhibit antitumor efficacy, mainly due to their repressive effects on angiogenesis. In addition, a recent non-clinical evaluation using mouse tumor models revealed that lenvatinib causes immunomodulatory effects, including activation of effector T-cells and regulation of tumor-associated macrophages (TAMs). Combined treatment with lenvatinib and anti-programmed cell death-1 antibody (anti-PD-1) resulted in enhanced antitumor activity relative to monotreatment with anti-PD-1 or lenvatinib. This review summarizes the antitumor mechanisms of lenvatinib and of lenvatinib plus anti-PD-1 combination therapy.

## Introduction

Tumor angiogenesis is crucial for the proliferation, invasion, and metastasis of cancer cells by providing nutrients and oxygen via blood circulation, to escape harsh tumor microenvironments, such as hypoxic conditions with low nutrients or pH [1–3]. Angiogenesis is mainly induced by secreting angiogenesis-promoting factors from cancer cells, including vascular endothelial growth factor (VEGF), fibroblast growth factor (FGF), angiopoietin, platelet derived growth factor (PDGF), and transforming growth factor-β. VEGF binds to the VEGF receptor (VEGFR) 2 on endothelial cells and induces differentiation of endothelial cells into tip cells, which leads to the branching of new blood vessels as well as the proliferation of endothelial cells. VEGF is also associated with the migration and activation of monocytes and macrophages via VEGFR1 [4].

Anti-angiogenic therapy for cancer treatment was initially proposed by Folkman, and a number of angiogenesis inhibitors have since been discovered [5]. In 2004, bevacizumab, a humanized anti-VEGF antibody, was approved for the treatment of advanced colorectal carcinoma [6]. Based on the antitumor efficacy of bevacizumab in humans and the essential role of VEGF in tumor angiogenesis in mouse tumor models, VEGFR signaling is considered a key pathway in tumor angiogenesis [7]. The efficacy of several angiogenesis inhibitors has been confirmed in other cancers, including advanced lung adenocarcinoma, renal cell carcinoma (RCC), and glioma [8].

Programmed cell death-1 (PD-1) was first identified as a gene that induces apoptosis in 1992 [9]. However, subsequent analysis of a spontaneous lupus-like autoimmune disease in PD-1 deficient mice revealed that PD-1 is expressed on activated T and B cells in the periphery, and on thymocytes, and negatively regulates the immune responses of these immune cell populations [10]. PD-L1 and PD-L2 are PD-1 ligands expressed in a variety of cancers as well as normal tissues. The expression of PD-L1 is upregulated by various stimuli, including interferon (IFN)-γ secreted by activated immune cells [10]. PD-L1 negatively regulates the cytolytic activity of CD8^+^ T-cells and induces immune tolerance against cancer. The finding that anti-PD-1 or anti-PD-L1 antibodies block the interaction between PD-1 and PD-Ls and restore the activation of CD8^+^ T-cells against cancer cells was clinically important [11]. Thus, humanized anti-PD-1 antibodies, including pembrolizumab, have been developed and were approved as immune checkpoint inhibitor treatments for several cancers, including unresectable and metastatic malignant melanoma, non-small cell lung cancer, unresectable urothelial cancer, and refractory non-Hodgkin lymphoma [12–14]. Although pembrolizumab provides durable antitumor activity in several cancer types, including a complete response in some patients, even after discontinuation [15–17], the efficacy of anti-PD-1 antibody monotherapy is restricted to approximately 20–30% of treated patients in many clinical studies [14]. Considering the proportion of patients who do not benefit from anti-PD-1 antibody treatment, means of improving anti-PD-1 treatment efficacy have been explored, such as combination treatments with other immune checkpoint inhibitors, including cytotoxic T-lymphocyte antigen 4 (CTLA-4) or lymphocyte-activation gene 3 (LAG3), or with other standard treatments, including chemotherapy, radiation, and molecular targeted therapy. Among these, angiogenesis inhibitors have also been explored for combination with immune checkpoint inhibitors [18].

This review focuses on the anti-angiogenic and immunostimulatory effects of lenvatinib, as well as the mechanism of action of combination therapy with lenvatinib and anti-PD-1 antibody.

## Lenvatinib: a multi-targeted tyrosine kinase inhibitor that mainly inhibits VEGF and FGF signals

Lenvatinib is an oral small molecule inhibitor of multiple receptor tyrosine kinases that targets VEGFR1–3, FGFR1–4, and other tyrosine kinases, including PDGFRα, KIT, and RET. Lenvatinib is approved as a monotherapy for patients with radioiodine-refractory differentiated thyroid cancer [19]; unresectable hepatocellular carcinoma (HCC) [20]; unresectable thymic cancer [21]. Lenvatinib is also approved for the patients with advanced renal cell carcinoma (RCC) in combination with everolimus, an mTOR inhibitor [22] and in combination with pembrolizumab, a humanized anti-PD-1 blocking antibody [23], and advanced endometrial cancer (EC) in combination with pembrolizumab [23, 24] (Table 1).

**Table 1:** Approved indications of monotherapies and combination therapies for lenvatinib

Indication	Study	Treatment	Country	Reference
Locally recurrent or metastatic, progressive, radioactive iodine-refractory differentiated thyroid cancer	SELECT study	Monotherapy	U.S., Europe, Japan, and other countries	Schlumberger et al. [19]
Unresectable hepatocellular carcinoma	REFLECT study	Monotherapy	U.S., Europe, Japan, and other countries	Kudo et al. [20]
Unresectable thymic carcinoma	REMORA study / NCCH1508	Monotherapy	Japan	Sato et al. [21]
Advanced renal cell carcinoma following one prior anti-angiogenic therapy	Study 205	Combination with Everolimus	U.S., Europe	Motzer et al. [22]
Advanced endometrial carcinoma that is mismatch repair proficient, or not microsatellite instability-high, who have disease progression following prior systemic therapy	Study 309 / KEYNOTE-775	Combination with Pembrolizumab	U.S., Europe, Japan, and other countries	Makker et al. [24]
Advanced renal cell carcinoma	CLEAR study / Study 307 / KEYNOTE-581	Combination with Pembrolizumab	U.S., Europe, Japan, and other countries	Motzer et al. [23]

In both in vitro and in vivo functional assays, lenvatinib demonstrates antitumor efficacy through anti-angiogenic activity [25]. In a VEGF-overexpressing KP-1 human pancreatic cancer xenograft model, lenvatinib inhibits VEGF-driven tumor growth associated with angiogenesis inhibition [25]. In a human cancer xenograft model panel, the sensitivity of antitumor activity by lenvatinib correlates with microvessel density in tumors [25].

It is noteworthy that lenvatinib suppresses VEGF- and FGF-induced angiogenesis. For example, lenvatinib inhibits VEGF- and FGF-stimulated tube formation in human umbilical vein endothelial cells (HUVECs) in an in vitro tube formation assay and lenvatinib significantly reduces VEGF- and FGF-induced tumor vasculature formation in an in vivo dorsal air sac assay [25]. In a mouse model inoculated with VEGFR-Fc-overexpressing cancer cells, continuous exposure to VEGFR-Fc induces expression of FGF ligands, while activation of FGFR signaling led to resistance to VEGFR inhibition [26]. However, lenvatinib shows antitumor efficacy in VEGFR therapy-resistant models, based on its dual inhibitory activity against VEGF and FGF signaling [26].

Thus, among many VEGF inhibitors, the dual inhibitory activity of lenvatinib against VEGF and FGF signaling is a distinctive feature of lenvatinib.

## Immune-suppressive function of VEGF

In addition to its critical role in tumor angiogenesis, VEGF is known to create a tumor immunosuppressive microenvironment in tumors. VEGFR is expressed in T-cells, including CD4^+^ T-cells, CD8^+^ T-cells, regulatory T-cells (Tregs), and myeloid cells, including dendritic cells (DCs), tumor-associated macrophages (TAMs), and myeloid-derived suppressor cells (MDSCs) as well as endothelial cells. VEGFR signaling in these immune cell populations suppresses antitumor immune response [27].

### CD8^+^ T-cells

CD8^+^ T-cells express low levels of VEGFR2, and activation of T-cell receptor (TCR) signaling upregulates VEGFR1 and VEGFR2 [28]. VEGF-A (VEGFA) induces the exhaustion of activated CD8^+^ T-cells by enhancing the expression of immune checkpoint molecules, including PD-1, CTLA-4, and LAG-3, and anti-VEGFA antibody decreases the expression of immune checkpoint molecules which lead to suppression of tumor growth in anti-PD-1 antibody-resistant tumor models [28].

### Tregs

VEGFR2 is selectively expressed on human FOXP3 high Tregs among CD4^+^ T-cells in tumor [29] and VEGFA induces the recruitment and proliferation of Tregs via activation of VEGFR2 signaling [30, 31]. Intratumoral FOXP3^+^VEGFR2^+^ Tregs are correlated with poor survival and disease-free survival in colorectal cancer patients [32]. Anti-VEGFA antibody treatment suppresses proliferation of Tregs in the serum of the CT26 mouse CRC model, and this result was confirmed in peripheral blood from CRC patients treated with anti-human VEGFA antibody, bevacizumab, in clinic. [31].

### DCs

Binding of VEGFA to VEGFR1 impairs the differentiation of hematopoietic progenitor cells (HPCs) into functional DCs [33]. VEGFA and impaired DC population is correlated in cancer patients and anti-VEGFA treatment is associated with decrease in accumulation of immature DC cells [34, 35]. This indicates that VEGFA suppresses normal differentiation of DC and impaired anticancer immunity. VEGFR2 is also highly expressed in plasmacytoid DCs (pDCs) and contributes to their homeostasis and function [36]. In this study, Agudo et al. showed that IFN-α is secreted from pDCs in response to pathogens and depletion of VEGFR2 significantly reduces IFN-α expression in pDCs treated with CpG [36]. This indicates that VEGFR2 signaling also plays important roles in regulation of innate immunity.

### Monocytes and macrophages

Several cytokines, chemokines, and growth factors are related to the migration of monocytes from bone marrow into tumors, including chemokines, colony stimulation factor (CSF)1, Tumor necrosis factor (TNF)-α, and VEGFA [37]. Monocytes and macrophages express VEGFR1 strongly, whereas expression of VEGFR2 is limited in a subpopulation of TAMs. VEGFA is mainly produced by cancer cells; however, subpopulations of TAMs secrete VEGFA to form microvessels in tumors [38]. VEGFA–VEGFR1 signaling induces the migration of monocytes from the bone marrow and VEGFR1^+^ macrophages contribute to local tissue angiogenesis [39]. VEGFR1 signaling in metastasis-associated macrophages is also critical for breast cancer metastasis. Additionally, VEGFR1 signaling in TAMs contributes to infiltration of TAMs into tumors, mainly into poorly vascularized areas of the tumor [40].

## Immunomodulatory activity of lenvatinib and combination treatment with anti-PD-1 antibody

Transcriptomic analysis of tumor tissues derived from cancer patients treated with pembrolizumab monotherapy in nine clinical trials identified 11 signatures associated with anti-PD-1 monotherapy response [41]. Among 11 signatures, 3 gene signatures were negatively associated with response to pembrolizumab monotherapy; one of these was an angiogenesis signature [41]. Considering the role of VEGFA in tumor immunity, we hypothesized that angiogenesis inhibitors, including lenvatinib, would change the tumor microenvironment to an immunostimulatory phenotype by affecting immunosuppressive cells, such as TAMs.

First, we investigated whether the antitumor activity of lenvatinib was associated with immune cell activation. Therefore, we examined the antitumor activity of lenvatinib in the Hepa 1–6 mouse HCC and CT26 mouse colon carcinoma models using athymic nude and immunocompetent mice. Interestingly, the antitumor activity of lenvatinib was more efficient in immune-competent mouse tumor models than in athymic nude mouse tumor models, suggesting that lenvatinib is more potent in immune-competent tumor microenvironments, including within T-cell populations [42, 43].

Next, we examined whether CD8^+^ T-cells were associated with the antitumor activity of lenvatinib in the Hepa 1–6 model. Using an anti-CD8 antibody, a CD8^+^ T-cell-depleted mouse model was established, and the antitumor activity of lenvatinib was evaluated. The result showed that CD8^+^ T-cell depletion partially diminished the antitumor activity of lenvatinib. These data suggested that this antitumor activity was complemented by the activation of CD8^+^ T-cells. In addition to the immune-stimulatory effect of lenvatinib on CD8^+^ T-cells, Zhang et al. reported that lenvatinib promoted tumor infiltration of natural killer (NK) cells by inducing adhesion molecules on both endothelial and NK cells and chemokines from cancer cells, including CXCL9 and CXCL10, which lead to tumor infiltration and activation of NK cells [44].

Based on these data, we investigated the antitumor activity of lenvatinib together with an anti-PD-1 in the Hepa 1–6 model. We showed that the combination treatment significantly inhibited tumor growth as compared to each individual treatment, although the antitumor activity of lenvatinib or anti-PD-1 treatment alone was more effective than non-treatment [43]. In the same experiment, it was noteworthy that there were some mice with non-palpable size tumors in the combination group. The antitumor activities of combined treatment were observed in other mouse tumor models, such as the CT26 and MC38 mouse colon carcinoma models, RAG and RENCA mouse RCC tumor models, B16F10 mouse melanoma model, MBT-2 and MB49 mouse bladder cancer models, and EMT6 mouse breast cancer model [42, 45, 46]. In a mouse syngeneic tumor model panel, lenvatinib plus anti-PD-1 sensitive models showed higher values of an immune-related gene signature at baseline compared with models that did not demonstrate benefit of the combination [46]. These data indicated that lenvatinib enhanced the antitumor activity of anti-PD-1.

Indeed, a phase Ib/II study of lenvatinib in combination with pembrolizumab in selected advanced solid tumor cohorts, including endometrial cancer, demonstrated promising antitumor activity [47].

## Mechanism of action of lenvatinib plus anti-PD-1 antibody combination treatment in antitumor immunity

To analyze the mechanism of action underlying the antitumor effect of the lenvatinib plus anti-PD-1 combination treatment in the Hepa 1–6 model, single-cell RNA-seq analysis was performed using CD45^+^ immune cells from Hepa 1–6 tumors [43]. Using specific immune cell markers, clusters of immune cell populations were divided into nine subpopulations, and the percentage change induced by treatment was examined in each immune cell population. Among these cell populations, “effector CD8^+^ T-cells” and “early activated CD8^+^ T-cells” were increased by each one of the individual treatments, while combination treatment further increased CD8^+^ T-cells as compared with single treatments. In contrast, lenvatinib and the combination treatment clearly decreased the monocyte and macrophage populations.

Next, we examined changes in the immune cell population of macrophages and CD8^+^ T-cells in flow cytometry analysis [42]. In macrophage populations, lenvatinib decreased TAM numbers, and the combination treatment increased M1 immunostimulatory macrophages, but decreased M2 immunosuppressive macrophages, thereby increasing the M1/M2 ratio. In T-cell populations, combination treatment increased CD8^+^ T-cells, particularly activated CD8^+^ T-cells secreting IFN-γ, perforin (PFN), and granzyme B (GZMB), although lenvatinib alone also significantly increased IFN-γ secreting CD8^+^ T-cells.

The effect of lenvatinib on macrophage polarization and was investigated in patients with HCC [48]. In flow cytometry analysis using peripheral blood mononuclear cells collected from patients with HCC, in CD14^+^ myeloid cells, expression of the M2 markers, CD206 and CD163, was strongly reduced after administration of lenvatinib compared with the M1 marker, CD86 [49]. The ratio of CD163^+^ M2 macrophages in CD68^+^ cells decreased after 4 weeks administration of lenvatinib in tumors from patients with HCC in immunohistochemical analysis [48].

In an ex vivo co-culture assay, the immunosuppressive function of TAMs against T-cell activation was confirmed [45]. T-cells and F4/80^+^ macrophages were purified from non-treatment RAG mouse RCC tumors. The proliferation of CMFDA-labeled T-cells was analyzed using flow cytometry. As a result, the proliferation of both CD8^+^ and CD4^+^ T-cells was suppressed in co-culture with F4/80^+^ myeloid cells in a cell number-dependent manner [45]. These data suggested that TAMs suppress T-cell activation. VEGF decreased trafficking and effector functions of T-cells, such as IFN-γ secretion and cytolysis, and increased the number of TAMs [50]. Thus, lenvatinib exhibits immunomodulatory activity in tumors, and it is indicated that many of these activities are derived from its VEGF-inhibitory activity.

## Immune modulatory effect of FGF signal inhibition by lenvatinib

FGF signal inhibitory activity is a unique feature of lenvatinib, in addition to its VEGF-inhibitory activity. FGF signaling plays an important role in the survival of cancer cells under hypoxic and low glycolytic metabolic conditions [51]. However, the relationship between FGF signaling and antitumor immunity remains unknown. FGF signaling has been reported to have an antagonistic effect on IFN signaling in keratinocytes during infection [52]. On this basis, the effect of lenvatinib on crosstalk between IFN-γ and FGF signal was investigated using the RAG mouse RCC tumor cells [45]. IFN-γ was shown to induce phosphorylation of STAT1 and to activate IFN signaling, leading to upregulation of IFN-γ downstream molecules, such as β2 macroglobulin (B2M) which is a component of MHC class-I; PD-L1; and chemokines, such as CXCL9, CXCL10, and CXCL11, which induce infiltration of T-cells into tumors. In RAG cells, under IFN-γ signal activation, FGF signal activation clearly inhibited the expression of IFN-γ downstream molecules, although it upregulated SOCS1 and suppressed phosphorylated STAT1. However, lenvatinib treatment suppressed downregulation of B2M and PD-L1 expression. These data suggested a crosstalk between IFN signaling and FGF signaling in RAG cells, and that FGF inhibition by lenvatinib can maintain the expression level of B2M and PD-L1 on cancer cells by prolonging IFN-γ signaling.

Additionally, lenvatinib directly induced cancer cell death due to its inhibitory effects on tyrosine kinases such as FGFR, which is associated with the release of damage-associated molecular patterns, including HMGB1, from dying cancer cells, known as immunogenic cell death (ICD) [53]. ICD induction increases PD-L1 expression by activating TLR4 signaling in cancer cells.

Thus, it is expected that direct effect of lenvatinib on cancer cells also enhanced the antitumor efficacy of anti-PD-1.

## Future perspectives

Lenvatinib is approved for patients with radioiodine-refractory differentiated thyroid cancer [19]; unresectable HCC [20]; thymic cancer [21]; advanced RCC in combination with everolimus [22] and in combination with pembrolizumab [23], and EC in combination with pembrolizumab [24] (Table 1). However, some patients do not benefit from these treatments, so further investigation of additional resistance mechanisms is needed. Using a CRISPR–Cas9 genetic screen, Jin et al. identified that EGFR is a lenvatinib-resistance factor in patients with HCC [54]. In HCC cell lines, the inhibition of EGFR induces synthetic lethality with lenvatinib in vitro. Additionally, treatment with lenvatinib plus gefitinib, an EGFR inhibitor, in 12 lenvatinib-unresponsive patients with advanced HCC resulted in four confirmed partial responses and four stable diseases. Hypoxia is induced by angiogenesis inhibitors, leading to expression of HIF-1/2 signaling downstream molecules which can escape cell death under severe tumor microenvironment condition. To achieve a synergistic effect with lenvatinib, combination treatment of lenvatinib plus the HIF-2α inhibitor belzutifan (MK-6482) with or without pembrolizumab is being tested in patients with RCC [55, 56]. Thus, several additional approaches may be further investigated in patients with tumors that are resistance to lenvatinib or lenvatinib plus pembrolizumab.

## Conclusions

**Fig. 1:**
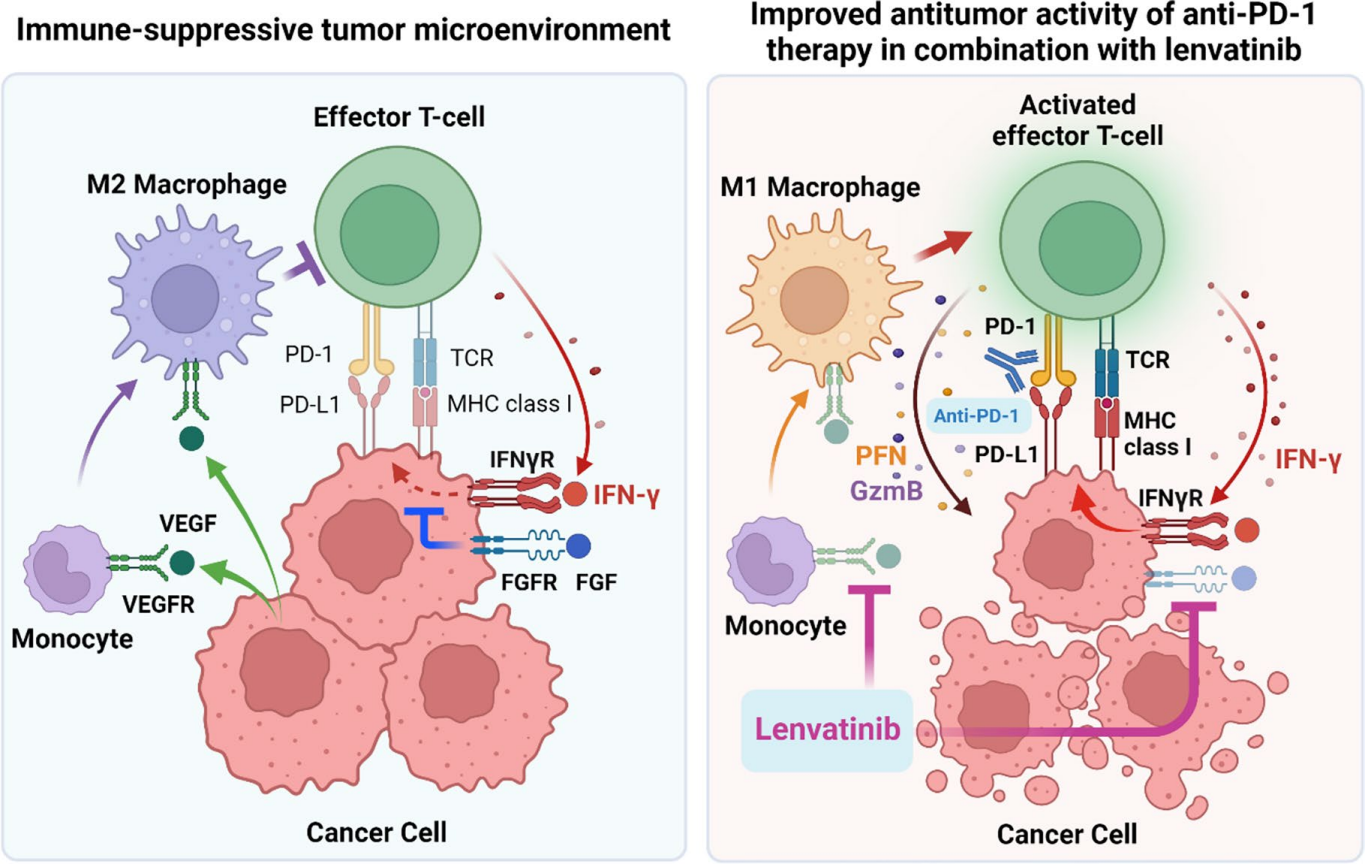
Mechanism of action of lenvatinib plus anti-PD-1 (Created in BioRender)

Lenvatinib is a multi-kinase inhibitor that inhibits VEGF and FGF signaling and that demonstrates antitumor efficacy through angiogenesis inhibition. Additionally, lenvatinib was recently found to possess immunomodulatory activity and to enhance the antitumor activity of anti-PD-1 treatment in mouse tumor models. Mechanistically, lenvatinib stimulated effector T-cell functions by regulating TAMs, and prolonged IFN-γ signaling by inhibiting FGF signaling in tumors (Fig. 1). Lenvatinib is approved and utilized for patients with several types of cancer, including thyroid cancer, HCC, and thymic cancer as monotherapy, and RCC and EC in combination with pembrolizumab. Considering the compelling efficacy observed in the clinical studies supporting these approvals, further study of lenvatinib in combination with other anticancer therapies with mechanisms relevant to overcome resistance is warranted.
